# Radioactive Molecules 2019–2020

**DOI:** 10.3390/molecules26030529

**Published:** 2021-01-20

**Authors:** Svend Borup Jensen

**Affiliations:** 1 Department of Nuclear Medicine, Aalborg University Hospital, 9000 Aalborg, Denmark; svbj@rn.dk; 2 Department of Chemistry and Biochemistry, Aalborg University, 9220 Aalborg, Denmark

It is with great pleasure that I have accepted the challenge of reviewing and summarizing the articles published in Molecules through 2019 and 2020 on radioactive molecules. This is a very interesting subject but also a challenging one, because research on radioactive molecules is a very versatile area. The progress in one area often is dependent on the further development and progression in others. For example the invention and commercializing of the SPECT, SPECT/CT, PET and PET/CT scanners, the invention of new useful tracer like [^18^F]FDG and [^68^Ga]Ga-PSMA as well as when the generators and cyclotrons were made commercially available, all are of inventions which have had a big impact on the entire area, this reflected on publications and publications rate.

A search in Molecules from 2019 to 2020 using the keyword “radio *” resulted in 181. A review of the 181 publications revealed that 109 had radioactive molecules in focus. Radioactive molecules are mainly used in medicine for diagnosing or treating of diseases. Six of the 109 publications had other focuses, e.g., the use of radiation for sterilization or preventing and/or cleaning up radioactive contamination. The further focus of the review will be on the 103 publications which, in some way, use or aim at using radioactive molecules for medical purpose. Of these 103 publications, 21 were reviews. To illustrate the diversity of the articles, an examination was made of the title, keywords, abstract and in a few cases, also, the content of the paper to find out whether the publications dealt with, diagnostics, therapy, synthesis and/or nuclide preparation. In addition, the nuclides referred to in the papers are also listed (Table 1, An overview of what topics and nuclides the articles deal with). 

None of the isotopes applied in the 103 above mentioned articles are naturally occurring so the first step would be to produce them, this is reflected in some of the publications as they have their roots in nuclear chemistry i.e., how to produce the isotopes. Other articles are in the field of medicinal chemistry, they deal with purification of the isotopes and incorporation of the isotopes into molecules of biological interest. It is common synthetic reaction and analytic procedure which is applied. They however have to be fast due to the short half-life of the isotope. Moreover, the syntheses must be suitable for automatization because protection to the staff against continuous exposer to radioactivity is required hence the need for automatization of the synthesis. Radioactive molecules are normally developed with the aim in mind of diagnosing diseases and/or to treat them. Some of the 103 articles describe how to employ animal or human biology and pharmaceutical chemistry to test the radioactive tracers in biologically relevant settings. However, looking at the articles published in molecules one quite often see that articles are a nice mixture of some or all of the above-mentioned chemical disciplines, nuclear-, medicinal-, synthetic-, analytical, animal-, human- and/or pharmaceutical chemistry.

The articles often aimed at exploring diagnosing or treating a specific disease, this were also registered. Of the 103 articles, 39 where concerning cancer [1] (eight articles on prostate [6], four on brain tumor/glioblastoma [2], three on ovaria cancer, three on neuroendocrine (somatostatin), two on, each of, breast cancer, bone cancer or colon rectal carcinoma and one on gastric cancer, lung cancer [10], solid tumor or multiple myeloma). There were 18 articles which deals with different kind of brain disorder (six articles on Alzheimer’s disease [3,4], three on Parkinson [4], three on neurological disorders and one on, each of, serotonin transporters, synaptic vesicle protein, phosphodiesterase, mood disorder or opioid receptor). There were seven articles about inflammation/infection (three articles on infection, two on osteomyelitis [8] and one on inflammation or on CXCR4 expression). Finally, there were nine articles on other diseases (two articles on cardiovascular and one on, each of, hyperlipidemia, peptide transporter, atherosclerotic plaques, sentinel node, invasive fungal, renal protection or vitamin E’s bio-distributions).

The clinical use as well as the research interest in the area of radioactive molecules have grown intensely though-out many years. This tendency is also seen in Molecules where the number of papers published in 2019–2020 is about the same as the numbers published in the previous 19 years (2000–2018).

I was asked to select ten papers which I find extra ordinarily interesting. It has not been easy because I had to reduce my originally shortlist from 37. To reduce further, I have tried to choose papers that support what I have tried to say in my mini-review on radioactive molecules that was published in Molecules in 2019 and 2020, namely that the research area “radioactive molecules” is a very versatile one.

## Figures and Tables

**Table 1:** An overview of what topics and nuclides the articles deal with.

	Diagnostic	Therapy	Synthesis	Nuclide Preparation	Ref.
	70.9%	23.3%	39.8%	8.7%	
^18^F	31		19		[1,2,3,4,5,6]
^99m^Tc	15		13		[1,6,7]
^68^Ga	12		9	1	[1,5,6,8,9]
^11^C	6		5		[2,4,6]
^125^I	5		2		[6,10]
^89^Zr	5		3	2	[2,6]
^15^O	4				[2]
^111^In	3				[1]
^64^Cu	2		3		[2,6]
^45^Ti	1		1	1	
^44^Sc	1			2	[8]
^55^Co	1				
^123^I	2				[10]
^3^H			1		
^177^Lu		7	6	1	[1,6,8]
^225^Ac		3	2	2	[6]
Alpha emitter		3			
^131^I		2			
^213^Bi		1			
^188^Re		1			
^212^Pb		1			
^111^Ag		1		1	
^211^At		1		1	
^105^Ru		1		1	
^161^Tb				1	[8]
^90^Sr				1	
